# Addition of Reduced Glutathione (GSH) to Freezing Medium Reduces Intracellular ROS Levels in Donkey Sperm

**DOI:** 10.3390/vetsci8120302

**Published:** 2021-12-02

**Authors:** Iván Yánez-Ortiz, Jaime Catalán, Ariadna Delgado-Bermúdez, Augusto Carluccio, Jordi Miró, Marc Yeste

**Affiliations:** 1 Equine Reproduction Service, Department of Animal Medicine and Surgery, Faculty of Veterinary Sciences, Autonomous University of Barcelona, ES-08193 Barcelona, Cerdanyola del Vallès, Spain; ivan.yanez22@gmail.com (I.Y.-O.); dr.jcatalan@gmail.com (J.C.); 2 Biotechnology of Animal and Human Reproduction (TechnoSperm), Institute of Food and Agricultural Technology, University of Girona, ES-17003 Girona, Spain; ariadna.delgado@udg.edu; 3 Unit of Cell Biology, Department of Biology, Faculty of Sciences, University of Girona, ES-17003 Girona, Spain; 4 Faculty of Veterinary Medicine, University of Teramo, Loc. Piano d’Accio, IT-64100 Teramo, Italy; acarluccio@unite.it

**Keywords:** sperm cryopreservation, reactive oxygen species (ROS), superoxide anion (O_2_^−^), reduced glutathione (GSH), antioxidants, donkey

## Abstract

In donkeys, the use of frozen-thawed sperm for artificial insemination (AI) leads to low fertility rates. Furthermore, donkey sperm produce a large amount of reactive oxygen species (ROS), and post-AI inflammation induces the formation of neutrophil extracellular traps (NETosis), which further generates many more ROS. These high ROS levels may induce lipid peroxidation in the sperm plasma membrane, thus affecting its integrity. Enzymatic and non-enzymatic antioxidants, mainly found in the seminal plasma (SP), are responsible for maintaining the redox balance. However, this fluid is removed prior to cryopreservation, thereby exposing sperm cells to further oxidative stress. The exogenous addition of antioxidants to the freezing medium can reduce the detrimental effects caused by ROS generation. Therefore, the aim of this study was to evaluate how the addition of different reduced glutathione (GSH) concentrations (control, 2 mM, 4 mM, 6 mM, 8 mM, and 10 mM) to fresh sperm affect their cryotolerance. Total and progressive motility, kinematic parameters and motile sperm subpopulations were significantly (*p* < 0.05) different from the control in treatments containing 8 mM and 10 mM GSH, but not at lower concentrations. Plasma and acrosome membrane integrity, mitochondrial membrane potential (MMP) and intracellular superoxide levels (O_2_^−^) were not affected (*p* > 0.05) by any GSH concentration. Interestingly, however, the addition of 8 mM or 10 mM GSH reduced (*p* < 0.05) the percentages of viable sperm with high overall ROS levels compared to the control. In conclusion, frozen-thawed donkey sperm are able to tolerate high GSH concentrations, which differs from what has been observed in other species. This antioxidant capacity suggests that ROS could be important during post-AI and that the impact of using exogenous antioxidants like GSH to improve the sperm resilience to freeze-thawing is limited in this species.

## 1. Introduction

The cryopreservation of genetic material is a milestone for reproductive biotechnology, not only in humans but also in other vertebrates [1], since it may be applied together with other assisted reproductive technologies (ART) for the dissemination of genes of genetically superior animals [2], as well as for the conservation of biodiversity and the protection of endangered species [3]. This last aspect is very relevant in the donkey because the size of the population has suffered a significant decrease in recent years [4], which directly affects the genetic variability of the existing breeds [5]. Research in this species has focused its efforts on improving sperm cryopreservation protocols, precisely to try to safeguard endangered donkey breeds [6,7,8,9,10,11,12,13,14,15,16,17,18]. However, the use of cryopreserved donkey sperm has been limited by the low conception rates obtained when jennies are inseminated [7,8,19]. This, which contrasts with the reproductive performance of mares, is largely due to the physiological endometritis that occurs in the jenny after artificial insemination (AI) [20,21].

In addition to the above, cryopreservation is known to induce freezing injuries in sperm due to their exposure to thermal and osmotic stresses and their high sensitivity to an oxidative environment, which leads to subsequent lipid peroxidation in the plasma membrane [22]. In the particular case of donkey sperm, and in a similar fashion to other species, this occurs because the plasma membrane contains a large amount of polyunsaturated fatty acids esterified into phospholipids [23]. Oxidative stress can be defined as a consequence of the imbalance between the cellular antioxidant defense system and the production of reactive oxygen species (ROS). Physiologically, sperm release ROS as a by-product of their metabolism; these ROS are scavenged by enzymatic and non-enzymatic antioxidants which play an instrumental role in maintaining the redox balance [24]. Both types of antioxidants, whose function is to prevent oxidative damage, are present in sperm or in seminal plasma (SP) [25]. In this context, it is worth mentioning that Papas et al. [26] showed that the antioxidant capacity of the enzymes present in the SP is significantly higher in donkeys than in horses. In spite of this, the sperm cryopreservation protocols currently applied in donkeys include the elimination of SP by centrifugation [27]. This makes sperm more susceptible to functional damage with lethal consequences [28] and lead them to undergo structural injuries, mainly at the chromatin level, which has a significant impact on male fertility [29]. In addition, it must be considered that, in equids, the presence of sperm during AI causes a powerful endometrial reaction with a large influx of polymorphonuclear neutrophils (PMN) into the uterine lumen [20,30,31,32]. These polymorphonuclear neutrophils act mainly via degranulating/extruding their DNA and bactericidal molecules (histones and enzymes), thereby forming neutrophil extracellular traps (NETs) that capture sperm without killing them [33]. Therefore, sperm:PMN binding in donkey activates one of the defense mechanisms of polymorphonuclear neutrophils, NETosis [34], which releases a large amount of ROS. These ROS levels are much greater than those generated by sperm [35] and even by the polymorphonuclear neutrophils themselves [36].

An alternative to counteract the negative effect that ROS generates on sperm from different mammalian species, including the human [37], is the addition of reduced glutathione (GSH) to the preservation medium in both cooling [38,39,40,41] and freezing [42,43,44,45,46]. Reduced glutathione is one of the most relevant non-enzymatic antioxidants in mammalian cells, and most of its scavenging effect results from the interaction with glutathione reductase (GSR) and glutathione peroxidase (GPX) [47]. Reduced glutathione contains a sulfhydryl group (-SH) that protects the disulfide bonds between chromatin fibers from the oxidative damage caused by ROS, thus maintaining the sperm nuclear structure during cryopreservation [47,48,49].

Despite the antioxidant properties of GSH to maintain the redox balance during cryopreservation, GSH concentrations greater than 2.5 mM have been found to be toxic to horse sperm when added to the freezing extender, as there is a significant reduction in the motility, viability and plasma membrane integrity after thawing [43]. The same effect has been observed in pigs, as the addition of 1 mM GSH to the freezing medium results in greater protection of sperm function than higher concentrations [41]. In donkeys, there is only one study that investigated the effects of supplementing the freezing medium with GSH; however, only one concentration was tested [16]. Testing the resilience of donkey sperm to oxidative stress using different GSH concentrations could shed some light into the reproductive strategy of this species [50,51,52]. In addition, supplementing freezing media with GSH could improve donkey sperm function and survival after thawing, which would help sperm to fight against the oxidative environment they encounter when reaching the jenny uterus after AI [20]. Therefore, the objective of this study was to evaluate how different GSH concentrations added to the freezing medium affect the cryotolerance of donkey sperm.

## 2. Materials and Methods

### 2.1. Donkeys and Sperm Samples

A total of 12 ejaculates from 4 adult Catalonian jackasses (age: between 3 and 6 years old) were used. Animals were clinically healthy and of proven fertility (good fertility rates), and were maintained in individual paddocks at the Equine Reproduction Service, Autonomous University of Barcelona (Bellaterra, Cerdanyola del Vallès, Spain). This is a center for the collection of semen from equids that holds an EU approval (authorization number: ES09RS01E) and operates under rigorous protocols which include the control of the health and welfare of animals. Thus, it was not necessary to receive a specific approval from the Ethics Committee of the Autonomous University of Barcelona (Bellaterra, Cerdanyola del Vallès, Spain) to handle animals, since the four jackasses were semen donors. Complying with the sanitary guidelines established by the Council of the European Communities in Directive 82/894/EEC of 21 December 1982, animals were free of equine viral arteritis, equine infectious anemia and equine contagious metritis.

Sperm collection was performed on a regular schedule in the morning through an artificial vagina (Hannover model; Minitüb GmbH, Tiefenbach, Germany) with the use of an in-line nylon filter to remove the gel fraction. Once the ejaculate was obtained, it was immediately diluted 1:5 (*v*:*v*) in a skim milk-based extender [53], prewarmed to 37 °C. The quality of fresh sperm was checked through the analysis of concentration (Neubauer chamber; Paul Marienfeld GmbH & Co. KG, Lauda-Königshofen, Germany), motility (CASA system; see Section 2.5 for details), and viability and morphology (eosin-nigrosin staining; see Section 2.6 for details). All samples were confirmed to be above the standard thresholds before freezing: viable sperm ≥85%, morphologically normal sperm ≥70%, and total motile sperm ≥80%.

### 2.2. Experimental Design

Before cryopreservation, each diluted sperm sample was centrifuged at 600× *g* and 20 °C for 15 min (Medifriger BL-S; JP Selecta SA, Barcelona, Spain); the supernatant was discarded and the pellet was resuspended in a commercial freezing medium containing glycerol and methylformamide as permeable cryoprotectants (BotuCRIO; Botupharma Animal Biotechnology, Botucatu, Brazil). Subsequently, sperm concentration and viability were again analyzed, and the same freezing medium (BotuCRIO) was added to obtain a final concentration of 200 × 10^6^ viable sperm/mL (normalized in all cases). The final volume was divided into 6 aliquots of 3 mL each, and a different concentration of reduced glutathione (GSH; G4251; Saint Louis, MO, USA; final concentrations: 2 mM, 4 mM, 6 mM, 8 mM, and 10 mM) was added; the control was not supplemented with GSH. A total of 6 separate 0.5 mL plastic straws were filled with the content of each aliquot (sperm sample with its respective treatment). Each straw was manually labeled with the freezing date, jackass name and treatment as follows: T1 = sperm (control); T2 = sperm + 2 mM GSH; T3 = sperm + 4 mM GSH; T4 = sperm + 6 mM GSH; T5 = sperm + 8 mM GSH; and T6 = sperm + 10 mM GSH.

### 2.3. Sperm Cryopreservation

All straws were cryopreserved in parallel using an automatic controlled-rate freezer (Ice-Cube 14S; Minitüb GmbH, Tiefenbach, Germany). Cooling/freezing was carried out in 3 stages: (1) cooling from 20 °C to 5 °C for 60 min at a rate of −0.25 °C/min, (2) freezing from 5 °C to −90 °C for 20 min at a rate of −4.75 °C/min, and (3) freezing from −90 °C to −120 °C for 2.7 min at a rate of −11 °C/min. Finally, straws were plunged into liquid nitrogen at −196 °C and stored in appropriate tanks for their conservation until analysis.

### 2.4. Sperm Thawing

Frozen sperm straws were thawed by incubation at 37 °C for 30 s in a hot water bath. The content of each straw was poured into a 10-mL conical tube for post-thaw sperm analysis. For each treatment and sperm sample, one straw was used for the assessment of motility (CASA system; see Section 2.5 for more details) and viability (eosin-nigrosin staining; see Section 2.6 for more details), and the other two were utilized for the evaluation of sperm parameters through flow cytometry (see Section 2.7 for details).

### 2.5. Sperm Motility Analysis

Sperm motility was evaluated by means of a CASA-Mot module of the ISAS v1.2 system (Proiser R + D, Valencia, Spain) equipped with a high-resolution digital camera model MQ003MG-CM (Proiser R + D, Valencia, Spain) capable of capturing up to 100 frames per second (fps). Before doing so, the content of each straw was diluted 1:2 (*v*:*v*) in a cooling extender based on skim milk prewarmed to 38 °C [53,54]. Briefly, 2 µL of each sperm sample was placed into a reusable Spermtrack10 chamber (Spk 10; Proiser R + D, Valencia, Spain), prewarmed to 38 °C. A minimum of 500 spermatozoa were counted per analysis under a 10× negative phase contrast microscope model UOP200i (Proiser R + D, Valencia, Spain). Total (TM, %) and progressive sperm motility (PM, %) were evaluated together regarding the following kinematic parameters: curvilinear velocity (VCL, µm/s), straight line velocity (VSL, µm/s), average path velocity (VAP, µm/s), linearity coefficient (LIN = [VSL/VCL] × 100, %), straightness coefficient (STR = [VSL/VAP] × 100, %), wobble coefficient (WOB = [VAP/VCL] × 100, %), amplitude of lateral head displacement (ALH, µm), and beat-cross frequency (BCF, Hz). The CASA-Mot settings were those recommended by the manufacturer: particle area > 4 and <75 µm^2^, connectivity: 6, minimum number of images to calculate ALH: 10. VAP ≥ 10 µm/s and STR ≥ 75% were used as cut-off values for motile sperm and progressively motile sperm, respectively. Three technical replicates were examined.

### 2.6. Sperm Viability Analysis

Sperm viability was analyzed by eosin-nigrosin staining [55]. Briefly, 10 µL of the content of each sperm sample was placed on a slide which was previously preheated to 37 °C. Immediately afterwards, 10 µL of the eosin-nigrosin stain was placed onto the sperm sample and mixed; the mixture was smeared. A bright field light microscope (Carl Zeiss, Göttingen, Germany) at 1000× magnification using immersion oil objective was used to evaluate a minimum of 200 sperm/sample. The percentage of viable sperm (eosin negative) was recorded and three technical replicates were evaluated.

### 2.7. Evaluation of Sperm through Flow Cytometry

The sperm functional parameters evaluated through flow cytometry were plasma membrane integrity (SYBR-14/PI), acrosome membrane integrity (PNA-FITC/PI), mitochondrial membrane potential (MMP, JC-1), intracellular ROS levels (H_2_DCFDA/PI), and intracellular superoxides levels (HE/YO-PRO-1). Fluorochromes were purchased from Molecular Probes (Thermo Fisher Scientific, Waltham, MA, USA). Sperm concentration was adjusted to 1 × 10^6^ sperm/mL before staining. For each sample, a total of 10,000 events were analyzed, and 3 technical replicates were evaluated.

A CytoFLEX flow cytometer (Beckman Coulter Fullerton, CA, USA) was used with a sheath flow rate set at 10 µL/min. Samples were excited with an argon ion laser (488 nm) at a power of 50 mW. Cell diameter/volume was assessed through the Coulter principle. To exclude cell aggregates and debris, distributions of two different dot plots were used. Cell aggregates were excluded on the basis of forward scatter height (FSC-H) and altitude (FSC-A) dot plot distribution, whereas subcellular debris were excluded based on the distribution of FSC-A and side scatter altitude (SSC-A) dot plots.

A total of 3 different optical filters were used: FITC was used for the analysis of SYBR-14, PNA-FITC, JC-1 monomers (JC-1_mon_), DCF^+^ and YO-PRO-1 (band pass: 525–540 nm); PE for the analysis of JC-1 aggregates (JC-1_agg_) and E^+^ (band pass: 585–542 nm), and PC5.5 for PI analysis (band pass: 690–650 nm). Information about each event (FSC-A, FSC-H, SSC-A, FITC, PE, and PC5.5) was collected in xit files and quantification from each sperm population was obtained using the CytExpert analysis software (Beckman Coulter Fullerton, CA, USA). The analyses were performed following the recommendations of the International Society for the Advancement of Cytometry (ISAC) [56]. For each parameter, the corresponding mean and standard error of the mean (SEM) were calculated.

#### 2.7.1. Plasma Membrane Integrity Analysis (SYBR-14/PI)

The analysis of plasma membrane integrity was carried out following the protocol described by Garner and Johnson [57], adapted to donkey sperm, using the LIVE/DEAD sperm viability kit (SYBR-14/PI). Briefly, sperm samples were incubated in the dark at 38 °C with SYBR-14 (final concentration: 31.8 nM) for 10 min and PI (final concentration: 7.6 µM) for a further 5 min. Three sperm populations were identified: (1) sperm with an intact plasma membrane, green-stained (SYBR-14^+^/PI^−^), (2) sperm with a damaged plasma membrane, red-stained (SYBR-14^−^/PI^+^), and (3) sperm with a damaged plasma membrane, both green- and red-stained (SYBR-14^+^/PI^+^). The unstained sperm (SYBR-14^−^/PI^−^) were considered as non-sperm particles (debris) and were used to correct the data in the other evaluations. The spill over of SYBR-14 in the PC5.5 channel (8.34%) was compensated.

#### 2.7.2. Acrosome Membrane Integrity Analysis (PNA-FITC/PI)

The integrity of the acrosome membrane was evaluated following the protocol described by Rathi et al. [58] adapted to donkey sperm using PNA-FITC and PI. Briefly, sperm samples were incubated in the dark at 38 °C with FITC-conjugated PNA (final concentration: 1.17 µg/mL) and PI (final concentration: 5.6 µm) for 10 min. Four sperm populations were identified: (1) sperm with an intact plasma membrane (PNA-FITC^−^/PI^−^), (2) sperm with a damaged plasma membrane that presented an acrosomal membrane that could not be completely intact (PNA-FITC^+^/PI^+^), (3) sperm with a damaged plasma membrane and missing outer acrosomal membrane (PNA-FITC^−^/PI^+^), and (4) sperm with a damaged plasma membrane (PNA-FITC^+^/PI^−^). Data were not compensated.

#### 2.7.3. Mitochondrial Membrane Potential (MMP) Analysis (JC-1)

The MMP analysis was performed following the protocol described by Ortega-Ferrusola et al. [59] adapted to donkey sperm using JC-1 iodide (5.5′, 6,6′-tetrachloro-1,1′, 3,3′ tetraethylbenzimidazolylcarbocyanine). In brief, sperm samples were incubated in the dark at 38 °C for 30 min with JC-1 (final concentration: 750 nM). In the presence of low MMP, JC-1 molecules remain as green-fluorescent monomers (JC-1_mon_), whereas in the presence of high MMP, they form orange-fluorescent aggregates (JC-1_agg_). Two sperm populations were distinguished: (1) low MMP sperm (cells presenting a JC-1_mon_ fluorescence intensity higher than a JC-1_agg_ fluorescence intensity) and (2) high MMP sperm (cells presenting a JC-1_agg_ fluorescence intensity higher than a JC-1_mon_ fluorescence intensity). Data were not compensated. The fluorescence intensity of JC-1_mon_ and JC-1_agg_ was recorded in all sperm populations, and the ratio between JC-1_agg_ and JC-1_mon_ was calculated.

#### 2.7.4. Intracellular Reactive Oxygen Species (ROS) Levels Analysis: H_2_O_2_ (H_2_DCFDA/PI) and O_2_^−^ (HE/YO-PRO-1)

The analysis of the intracellular levels of ROS was carried out following the protocol described by Morrell et al. [60] and Guthrie and Welch [61], adapted to donkey sperm. The combination of 2′, 7′-dichlorodihydrofluorescein diacetate (H_2_DCFDA) and PI was used to measure overall intracellular ROS levels, and the combination of hydroetidine (HE) and YO-PRO-1 was used to measure intracellular levels of superoxide anion (O_2_^−^).

For ROS evaluation, sperm samples were incubated in the dark at 38 °C with H_2_DCFDA (final concentration: 50 µM) for 20 min; thereafter, PI (final concentration: 6 µM) was added and samples were incubated for 5 min. In the presence of ROS, H_2_DCFDA is de-esterified and oxidized into the highly fluorescent molecule DCF^+^. Therefore, four sperm populations were identified: (1) viable sperm with high ROS levels (DCF^+^/PI^−^), (2) non-viable sperm with high ROS levels (DCF^+^/PI^+^), (3) viable sperm with low ROS levels (DCF^−^/PI^−^), and (4) non-viable sperm with low ROS levels (DCF^−^/PI^+^). Data were not compensated. The fluorescence intensity of DCF^+^ was recorded in all sperm populations.

For the measurement of the intracellular levels of O_2_^−^, sperm samples were incubated in the dark at 38 °C with HE (final concentration: 5 µM) and with YO-PRO-1 (final concentration: 31.25 nM) for 30 min. In the presence of O_2_^−^, HE is oxidized into fluorescent ethidium (E^+^). This allowed the observation of four sperm populations: (1) viable sperm with high O_2_^−^ levels (E^+^/YO-PRO-1^−^), (2) non-viable sperm with high O_2_^−^ levels (E^+^/YO-PRO-1^+^), (3) viable sperm with low·O_2_^−^ levels (E^−^/YO-PRO-1^−^), and (4) non-viable sperm with low O_2_^−^ levels (E^−^/YO-PRO-1^+^). The spill over of E^+^ into the FITC channel (3.62%) was compensated. The fluorescence intensity of E^+^ was recorded in all sperm populations.

### 2.8. Statistical Analyses

Statistical analyses were performed with the R statistical package (V 4.0.3, R Core Team; Vienna, Austria) and graphs were plotted with GraphPad Prism (V 8.4.0, GraphPad Software LLC; San Diego, CA, USA). The Shapiro–Wilk test was run to verify the normal distribution of data and the Levene test checked the homoscedasticity. When necessary, data were transformed with arcsin √x to match with parametric assumptions. When these assumptions were not met, a non-parametric analysis was performed using the Kruskal–Wallis test followed by the Wilcoxon test (*p* ≤ 0.05) for pairwise comparisons. The effects of the different GSH concentrations (control, 2 mM, 4 mM, 6 mM, 8 mM, and 10 mM) on motility, viability (eosin-nigrosin), plasma membrane integrity (SYBR14^+^/PI^−^), acrosome membrane integrity (PNA-FITC^−^/PI^−^), MPP (JC-1_agg_), intracellular H_2_O_2_ levels (DCF^+^/PI^−^), and intracellular O_2_^−^ levels (E^+^/YO-PRO-1^−^), as well as the geometric mean of fluorescence intensity (GMFI) of JC-1_agg_, DCF^+^ and E^+^ after thawing, were analyzed using a one-way ANOVA, followed by the Bonferroni post-hoc test (*p* ≤ 0.05) for pairwise comparisons.

Motile sperm subpopulations were obtained by applying the procedure described by Martí et al. [62]. Briefly, a principal component analysis (PCA) was performed from the kinematic parameters (VCL, VSL, VAP, LIN, STR, WOB, ALH, and BCF) of each sperm cell after thawing. The matrix obtained was rotated using the Varimax method with Kaiser normalization, where each spermatozoon was assigned a regression score. These values were used to perform a non-hierarchical, multivariate cluster analysis using the *k*-means model based on Euclidean distances. Finally, the proportion of spermatozoa in each subpopulation was calculated to analyze the effects of the different GSH concentrations through a one-way ANOVA, followed by the Bonferroni post-hoc test (*p* ≤ 0.05) for pairwise comparisons.

In all analyses, the results are expressed as means ± SEM.

## 3. Results

### 3.1. Sperm Motility

**Figure 1:**
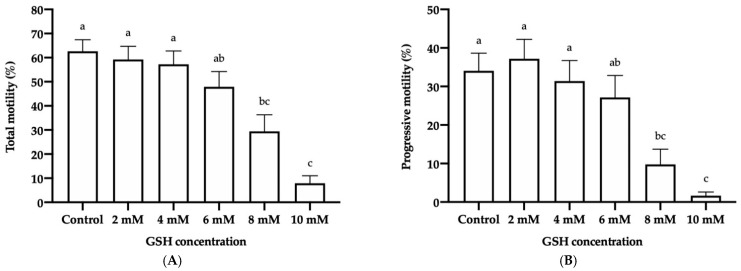
Mean ± SEM of the total (**A**) and progressive (**B**) motility of frozen-thawed donkey sperm following cryopreservation with different reduced glutathione (GSH) concentrations. (a–c) Different letters indicate significant differences (*p* ≤ 0.05) between the control and GSH treatments.

The percentage of total motile sperm at post-thaw (Figure 1A) was significantly lower in the treatments containing 8 mM GSH (29.36% ± 6.88%) and 10 mM GSH (7.85% ± 3.10%) than in the control (62.56% ± 4.81%) and those containing 2 mM GSH (59.18% ± 5.42%) and 4 mM GSH (57.20% ± 5.50%). The concentration of 6 mM GSH (47.81% ± 6.36%) only differed from that of 10 mM (*p* < 0.05). In addition, the percentage of progressively motile sperm (Figure 1B) decreased significantly when GSH concentrations of 8 mM (9.76% ± 3.95%) and 10 mM (1.65% ± 0.96%) were added, compared to the control (34.05% ± 4.61%), 2 mM GSH (37.20% ± 5.04%) and 4 mM GSH (31.41% ± 5.30%). The concentration of 6 mM (27.14% ± 5.70%) was only different from that of 10 mM (*p* < 0.05).

On the other hand, the kinematic parameters of frozen-thawed donkey sperm remained without significant changes at concentrations ≤ 6 mM GSH compared to the control (Table 1). Indeed, VCL, VAP, WOB and ALH were significantly lower in the treatment containing 10 mM than in the control (*p* < 0.05), whereas VSL, LIN, STR, and BCF were lower than the control in those having 8 mM and 10 mM GSH (*p* < 0.05).

### 3.2. Motile Sperm Subpopulations

Four motile sperm subpopulations were identified in frozen-thawed donkey sperm (Table 2). Subpopulation 1 (SP1) was characterized by being the fastest (higher values of VCL, VSL, and VAP) and most progressive (high values of LIN, STR, ALH and BCF, although WOB was intermediate). Subpopulation 2 (SP2) was the slowest and least progressive, with the lowest ALH and BCF values. Subpopulation 3 (SP3) presented intermediate velocity and progressivity values similar to those of SP1, with an intermediate ALH and a BCF similar to SP1. Finally, subpopulation 4 (SP4) was also characterized by presenting intermediate speed, progressivity values, ALH and BCF. Although these parameters were lower than in SP1 and SP3, SP4 exhibited the highest WOB.

**Figure 2:**
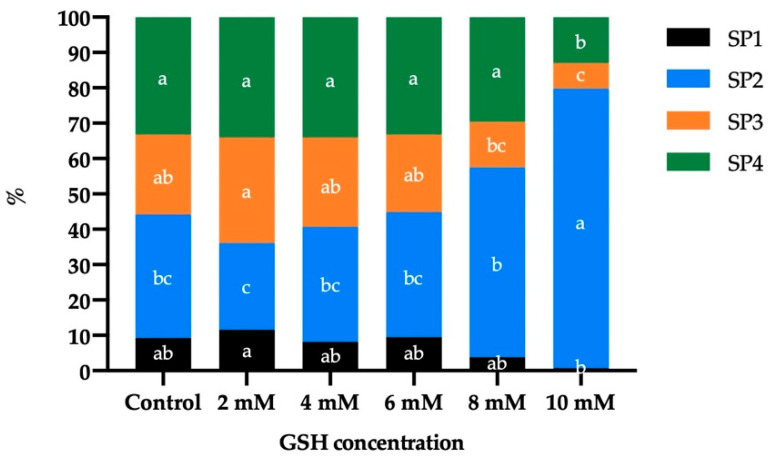
Distribution of the four motile sperm subpopulations (SP) identified in frozen-thawed donkey sperm following cryopreservation with different concentrations of reduced glutathione (GSH). (a–c) Different letters indicate significant differences (*p* ≤ 0.05) between the control and GSH treatments.

The proportions of motile spermatozoa of SP2 identified in frozen-thawed donkey sperm (Figure 2) were significantly higher in the presence of 8 mM (53.65% ± 7.10%) and 10 mM GSH (79.04% ± 9.01%) than in that of 2 mM GSH (24.56% ± 3.37%). On the contrary, SP3 reduced its proportion in the presence of 8 mM (12.88% ± 3.34%) and 10 mM GSH (7.24% ± 4.66%) compared to 2 mM GSH (29.87% ± 2.77%; *p* < 0.05). Similarly, the proportion of sperm belonging to SP1 was significantly lower in the treatment containing 10 mM GSH than in that with 2 mM GSH (0.72% ± 0.58% vs. 11.52% ± 2.28%), and the proportion of SP4-sperm was lower in the presence of 10 mM GSH (13.01% ± 4.92%) than in the control (34.05% ± 1.83%) and treatments with 2 mM (34.11% ± 1.42%), 4 mM (33.23% ± 1.94%), and 6 mM GSH (29.67% ± 3.83%).

### 3.3. Sperm Viability

**Figure 3:**
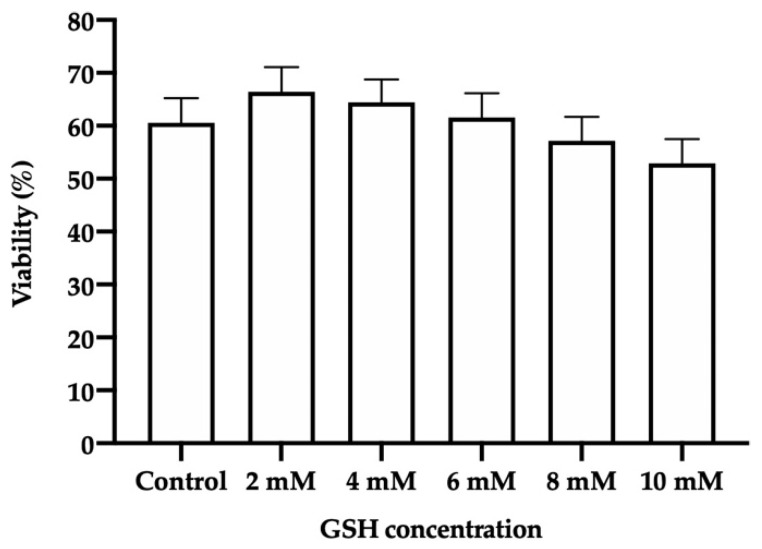
Mean ± SEM of the viability of frozen-thawed donkey sperm following cryopreservation with different reduced glutathione (GSH) concentrations. No significant differences (*p* ≤ 0.05) between the control and GSH treatments were found.

No significant differences in the viability of frozen-thawed donkey sperm (evaluated with eosin-nigrosin staining) between the different GSH concentrations and the control were found (Figure 3).

### 3.4. Sperm Quality

#### 3.4.1. Sperm Membrane Integrity

**Figure 4:**
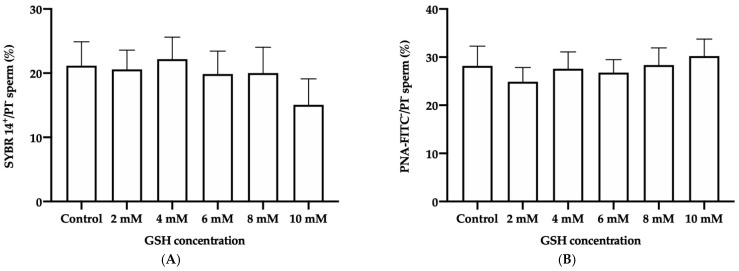
Mean ± SEM of the percentages of sperm with an intact plasma membrane (SYBR14^+^/PI^−^; (**A**)) and with an intact acrosome membrane (PNA-FITC^−^/PI^−^; (**B**)) observed in frozen-thawed donkey sperm after cryopreservation with different GSH concentrations. No significant differences (*p* ≤ 0.05) between the control and GSH treatments were found.

No significant differences in the percentage of frozen-thawed donkey sperm with intact plasma membrane (SYBR14^+^/PI^−^) were found between the control and the different GSH concentrations (Figure 4A).

#### 3.4.2. Acrosome Membrane Integrity

Similarly, no significant differences in the percentage of frozen-thawed donkey sperm with an intact acrosome membrane (PNA-FITC^−^/PI^−^) were observed between the different GSH concentrations and the control (Figure 4B).

#### 3.4.3. Mitochondrial Membrane Potential (MPP)

**Figure 5:**
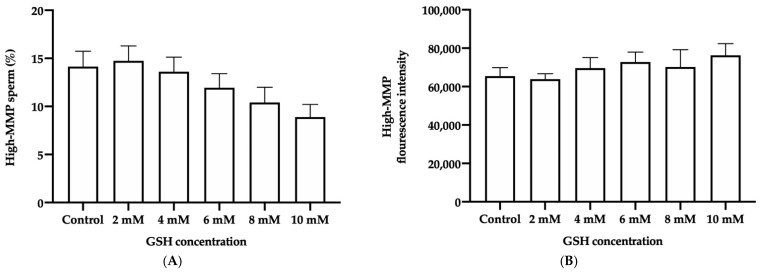
Mean ± SEM of the percentage of spermatozoa with high mitochondrial membrane potential (high-MMP; higher proportion of JC-1_agg_ than JC-1_mon_) (**A**) and geometric mean fluorescence intensity of JC-1_agg_ (GMFI, PE channel) in the sperm population with high-MMP (**B**) observed in frozen-thawed donkey sperm following cryopreservation with different GSH concentrations. No significant differences (*p* ≤ 0.05) between the control and GSH treatments were found.

No significant differences in the percentage of frozen-thawed donkey sperm with high-MMP (JC-1_agg_) were seen between the different GSH concentrations and the control (Figure 5A). Similarly, the GMFI of JC-1_agg_ in the high-MMP sperm population did not differ from the control when GSH was added to the freezing medium (Figure 5B).

#### 3.4.4. Intracellular Reactive Oxygen Species (ROS) Levels: H_2_O_2_ and O_2_^−^

**Figure 6:**
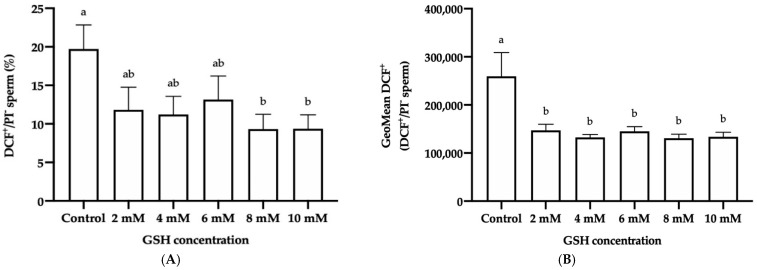

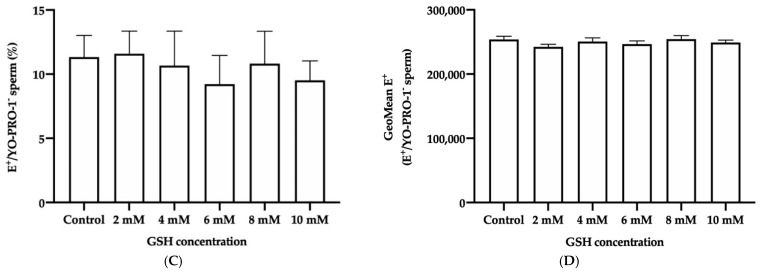
Mean ± SEM of the percentage of viable sperm with high overall ROS levels (DCF^+^/PI^−^, (**A**)), geometric mean fluorescence intensity of DCF^+^ (GMFI, FITC channel) in the DCF^+^/PI^−^ sperm population (**B**), percentage of viable spermatozoa with high·O_2_^−^ levels (E^+^/YO-PRO-1^−^, (**C**)), and geometric mean fluorescence intensity of E^+^ (GMFI, channel PE) in the E^+^/YO-PRO-1^−^ sperm population (**D**) observed in frozen-thawed donkey sperm following cryopreservation with different reduced glutathione (GSH) concentrations. (a,b) Different letters indicate significant differences (*p* ≤ 0.05) between the control and GSH concentrations in (**A**,**B**). No significant differences (*p* ≤ 0.05) between the control and GSH treatments in (**C**,**D**) were found.

The GSH concentrations of 8 mM and 10 mM significantly (*p* < 0.05) decreased the percentage of frozen-thawed donkey sperm with high ROS levels (DCF^+^/PI^−^) compared to the control (9.33% ± 1.91% and 9.36% ± 1.81% vs. 19.72% ± 3.13%, respectively; Figure 6A). Exposure to any GSH concentration also reduced (*p* < 0.05) the GMFI of DCF^+^ in the DCF^+^/PI^−^ sperm population (147,217.30 ± 12,735.15 in 2 mM GSH, 132,642.2 ± 5966.27 in 4 mM GSH, 145,301.20 ± 9617.71 in 6 mM GSH, 130,855.70 ± 8405.67 in 8 mM GSH, and 133,857.40 ± 9184.45 in 10 mM GSH) with respect to the control (259,537.70 ± 49,296.13; Figure 6B).

On the other hand, no significant differences in either the percentage of frozen-thawed donkey sperm with high·O_2_^−^ levels (E^+^/YO-PRO-1^−^) or the GMFI of E^+^ in the E^+^/YO-PRO-1^−^ sperm population were observed between the different GSH concentrations and the control (Figure 6C,D).

## 4. Discussion

The combination of the cellular component (sperm) produced by the germinal epithelium of the testes and the liquid component (SP) generated by the epididymis and the accessory sex glands [63,64] causes semen to become a complex redox system due to the antioxidant action exerted mostly by SP and, to a lesser extent, by sperm, as well as by the pro-oxidant action of sperm through ROS production [65]. This last action is more important during sperm cryopreservation in equids, because sperm cells lose the antioxidant support of SP before freezing. While intracellular GSH levels in horse sperm have been quantified [66], it is not clear whether this produced amount is high enough for sperm to have the ability to scavenge the oxidative stress generated by ROS and thus maintain redox balance without the antioxidant support of SP. In the case of donkeys, it has recently been shown that sperm are capable of tolerating high ROS concentrations (H_2_O_2_) and that in the presence of PMN, SP plays a decisive role in the regulation of the ROS produced during NETosis [36]. In this scenario, the addition of exogenous GSH could compensate in a certain way for the antioxidant component that sperm lose when cryopreserved.

The first hypothesis of this study envisaged that adding GSH to the freezing medium would increase the ability of donkey sperm to withstand cryopreservation. However, our results indicate that supplementation of the freezing medium with GSH at a concentration as high as 10 mM does not affect the viability, plasma and acrosome membrane integrity, and MMP of post-thaw sperm. However, sperm motility was reduced almost to zero. This surprising finding in the donkey provides us with valuable information to understand the reproductive strategy in this species, since the tolerance to GSH observed herein was much higher than that reported in horses [43], pigs [41], sheep [42], cattle [45], and dogs [44]. In effect, in those species, adding high concentrations of GSH to the freezing medium results in a significant decrease in all the aforementioned parameters of post-thaw sperm; in fact, in some cases, these high concentrations have even been reported to be cytotoxic [43].

Regarding the motility of frozen-thawed donkey sperm, we observed a significant effect of high GSH concentrations on TM and PM, which decreased in the presence of 8 mM (29.36% ± 6.88% and 9.76% ± 3.95%, respectively) and 10 mM GSH (7.85% ± 3.10% and 1.65% ± 0.96%, respectively) with respect to the control (62.56% ± 4.81% and 34.05% ± 4.61%, respectively). Interestingly, frozen-thawed donkey sperm were found to be more tolerant to exogenous GSH supplementation than their horse counterparts, as TM and PM in the latter are known to be reduced in the presence of 5 mM and 2.5 mM GSH, respectively. Similarly, VSL, LIN, STR, and BCF values were significantly reduced at 8 mM and 10 mM GSH, whereas the other kinematic parameters (VCL, VAP, WOB, and ALH) were affected in the presence of 10 mM GSH. In addition, in horse sperm, LIN, STR, and BCF are decreased in the presence of a GSH concentration ≥2.5 mM; furthermore, VSL and ALH are decreased at ≥5 mM GSH, and VCL and VAP are decreased at ≥7.5 mM GSH [43]. Moreover, four separate subpopulations were herein identified in frozen-thawed donkey sperm, which is in agreement with previous research in this species [27,50,67]. We found that the treatment containing 10 mM GSH significantly increased the proportion of sperm belonging to SP2 (the slowest and least progressive subpopulation), reduced those of sperm belonging to SP3 and SP4 (the subpopulations with intermediate kinematic parameters), and did not affect that of SP1 (the fastest and most progressive subpopulation) with respect to the control. Remarkably, 2 mM, 4 mM, and 6 mM GSH were found not to modify the structure of the frozen-thawed motile sperm subpopulations compared to the control. The analysis of sperm subpopulations once again highlighted the cryotolerance of frozen-thawed donkey sperm as, while the presence of 2 mM GSH is known to modify the structure of those subpopulations in pigs, increasing the fastest subpopulation and decreasing the slowest one [68], no changes were observed herein for concentrations ≤6 mM GSH. 

While sperm viability and integrities of plasma and acrosome membranes were not affected by the presence of high GSH concentrations, no treatment, even the ones containing low concentrations of this antioxidant, improved these sperm parameters post-thaw. This is contrary to what was found by Kumar et al. [16] in frozen-thawed sperm from exotic donkeys (Poitou breed, Martina Franca), where there was a significant increase in motility, viability and plasma membrane integrity when the freezing medium was supplemented with 2.5 mM. Our results, however, agree with the aforementioned study in the percentage of sperm with an intact acrosome membrane, since no differences in this parameter were observed in our study or in that of Kumar et al. [16], even when using two different techniques (flow cytometry vs. Giemsa stain + microscopy). Moreover, comparing our results with those found in other species may provide some clues, despite the fact that inconsistent data are seen in the literature. In cattle, Gangwar et al. [45] observed that adding 0.5 mM GSH to the freezing medium increased the viability, plasma and acrosome membrane integrity, and MMP of frozen-thawed sperm. In horses, Oliveira et al. [39] reported that a higher concentration of GSH (2.5 mM) was required to observe an improvement in the percentage of post-thaw sperm with an intact plasma membrane, despite the fact that acrosome integrity was similar to the control. In pigs, Estrada et al. [69] supplemented the freezing medium with 2 mM GSH and observed an increase in the percentages of viable and acrosome-intact sperm, and in fertilizing ability. On the contrary, Silva et al. [42] did not find any improvement in the quality (plasma and acrosome membrane integrity, and MMP) of frozen-thawed sheep sperm with 2 mM GSH in relation to the control, which was in agreement with our observations in the current study.

As previously mentioned, whereas TM and PM were found to decrease when 8 mM and 10 mM GSH were added to the freezing medium, viability, plasma and acrosome membrane integrity, and MMP did not differ from the control. Moreover, we observed that the percentage of viable sperm with high intracellular ROS levels was significantly lower in the treatments containing 8 mM and 10 mM GSH than in the control. These observations are in relative or slight agreement with our second hypothesis, which suggested that motility, viability, plasma and acrosome membrane integrity, MMP, and intracellular ROS levels of post-thaw sperm would be affected by the addition of GSH to the freezing medium. ROS production is known to alter the function and structure of sperm membranes [70], causing a detrimental effect on sperm motility and membrane integrity [22]. Our results indicate that the detrimental effects of ROS could be counteracted by the presence of GSH. However, the other parameters of sperm functionality did not improve with the presence of GSH. In fact, the results showed that GSH is effective in scavenging ROS up to 8 mM, and could have an effect on sperm motility. Further studies are needed to address whether fertility is also compromised or if other antioxidants are able to improve the functionality and fertility of donkey sperm. As the post-translational modification of protein thiols is the most likely mechanism to maintain redox balance [41,71], one could speculate that supplementing the freezing medium with exogenous GSH could reverse the oxidative stress generated during the freezing and thawing of donkey sperm. This could maintain the integrity of plasma and acrosome membranes.

On the other hand, it is important to highlight that GSH supplementation did not affect the production of intracellular O_2_^−^ levels during cryopreservation. Physiologically, O_2_^−^ is scavenged by superoxide dismutase (SOD) [72], another antioxidant enzyme present in donkey SP, rather than by the glutathione complex enzymes (GSR and GPX). Freeze-thawing reduces SOD activity in response to an increase in O_2_^−^ production [65]; this enzyme converts O_2_^−^ into H_2_O_2_ by dismutation [73]. This leads one to posit that if there was high O_2_^−^ production in the long run, there would be a greater amount of H_2_O_2_. This excess of H_2_O_2_ could be scavenged by exogenous GSH and thus reduce the percentage of sperm with high H_2_O_2_ levels, as reported herein.

Another important implication of our results is related to what happens in the jenny’s endometrium when AI is performed with frozen-thawed semen. In this scenario, donkey sperm are exposed to an oxidative environment in the uterus, which results from the production of ROS by PMN during the NETosis triggered by the presence of sperm [21]. Based on our results, GSH concentrations were not able to scavenge intracellular sperm ROS levels. Therefore, it seems reasonable to suggest that exogenous GSH supplementation between 2 mM and 6 mM could be a strategy against oxidative stress in this species after cryopreservation. This could be especially relevant after freeze-thawing if one bears in mind that SP, which is the main antioxidant source in semen, is removed before cryopreservation. However, one aspect that must be taken into account is that the degree of reduction in intracellular ROS levels observed at high GSH concentrations (8 mM and 10 mM) was not sufficient to positively affect sperm motility; in fact, it reduced sperm motility. This would not be in agreement with most observations in other mammalian species that indicate that sperm sensitivity to oxidative stress and cryogenic damage results in a reduction in motility after freeze-thawing [45,74,75]. In effect, we observed that, although high concentrations of GSH regulated the intracellular ROS levels, motility decreased. This opposite relationship advises that the impact of ROS on the other functional parameters in donkey sperm differs from other species.

## 5. Conclusions

According to the results obtained, frozen-thawed donkey sperm were found, on the one hand, to have a surprising capacity to tolerate high GSH concentrations compared to other species, maintaining viability, plasma membrane and acrosome integrity, and MMP. On the other hand, supplementation of the freezing medium with GSH appears to be necessary to control intracellular ROS levels, mainly H_2_O_2_, produced during freeze-thawing as well as post-AI, and may improve reproductive performance in the jenny. However, the highest GSH concentrations (8 mM and 10 mM) affected sperm motility, which suggests that how donkey sperm handle ROS generation differs from other species. Therefore, conducting in vivo studies is much warranted to complement the results obtained in this work.

## Figures and Tables

**Table 1:** Mean ± SEM of the kinematic parameters of frozen-thawed donkey sperm following cryopreservation with different reduced glutathione (GSH) concentrations.

Parameter	GSH Concentration					
	Control	2 mM	4 mM	6 mM	8 mM	10 mM
VCL (µm/s)	134.66 ± 8.53 ^a^	152.04 ± 6.74 ^a^	139.16 ± 7.43 ^a^	138.69 ± 9.47 ^a^	114.97 ± 13.76 ^a^	57.03 ± 18.80 ^b^
VSL (µm/s)	53.16 ± 4.55 ^a^	60.51 ± 4.09 ^a^	52.71 ± 4.11 ^a^	50.47 ± 5.20 ^a^	30.14 ± 4.76 ^b^	13.53 ± 5.08 ^b^
VAP (µm/s)	69.42 ± 4.75 ^a^	76.22 ± 3.75 ^a^	70.92 ± 2.98 ^a^	68.26 ± 4.51 ^a^	53.05 ± 5.95 ^a^	27.23 ± 8.54 ^b^
LIN (%)	37.02 ± 1.20 ^a^	37.96 ± 1.26 ^a^	35.59 ± 1.29 ^a^	33.96 ± 1.67 ^a^	23.06 ± 2.61 ^b^	10.98 ± 3.75 ^c^
STR (%)	70.07 ± 1.91 ^a^	74.07 ± 2.09 ^a^	68.06 ± 3.21 ^a^	67.37 ± 3.21 ^a^	47.72 ± 5.40 ^b^	23.19 ± 7.92 ^c^
WOB (%)	51.61 ± 0.39 ^a^	50.65 ± 0.65 ^a^	51.96 ± 1.50 ^a^	49.95 ± 0.88 ^a^	44.14 ± 4.51 ^a^	24.70 ± 7.55 ^b^
ALH (µm)	1.81 ± 0.08 ^a^	2.06 ± 0.08 ^a^	1.92 ± 0.10 ^a^	1.93 ± 0.11 ^a^	1.62 ± 0.18 ^a^	0.83 ± 0.27 ^b^
BCF (Hz)	28.23 ± 2.28 ^a^	29.27 ± 2.19 ^a^	25.84 ± 1.46 ^a^	23.57 ± 1.91 ^ab^	15.19 ± 1.90 ^bc^	8.05 ± 2.52 ^c^

VCL (µm/s): curvilinear velocity; VSL (µm/s): straight line velocity; VAP (µm/s): average path velocity; LIN (%): linearity coefficient; STR (%): straightness coefficient; WOB (%): wobble coefficient; ALH (µm): amplitude of lateral head displacement; BCF (Hz): beat-cross frequency. (^a–c^) Different letters indicate significant differences (*p* ≤ 0.05) between the control and GSH treatments.

**Table 2:** Structure of the four motile sperm subpopulations identified in frozen-thawed donkey sperm following cryopreservation with different reduced glutathione (GSH) concentrations.

Parameter	SP1		SP2		SP3		SP4	
	Mean ± SEM	Range	Mean ± SEM	Range	Mean ± SEM	Range	Mean ± SEM	Range
VCL (µm/s)	265.51 ± 0.81	197.90–429.30	69.32 ± 0.35	0.00–147.30	198.68 ± 0.35	127.50–276.00	134.67 ± 0.32	66.50–218.70
VSL (µm/s)	115.39 ± 0.54	10.80–191.30	15.79 ± 0.13	0.00–54.70	83.56 ± 0.30	3.70–152.10	49.42 ± 0.22	1.80–105.00
VAP (µm/s)	131.57 ± 0.40	78.60–216.50	33.31 ± 0.19	0.00–91.90	99.91 ± 0.22	44.30–165.90	69.05 ± 0.17	25.10–123.90
LIN (%)	44.13 ± 0.23	3.90–75.30	24.06 ± 0.19	0.00–92.30	42.75 ± 0.17	1.50–95.60	38.03 ± 0.19	1.20–100.00
STR (%)	87.42 ± 0.27	7.70–100.00	48.01 ± 0.29	0.00–99.20	83.59 ± 0.23	3.70–100.00	71.71 ± 0.25	2.30–100.00
WOB (%)	50.09 ± 0.18	27.00–85.30	48.88 ± 0.17	0.00–100.00	50.80 ± 0.13	19.20–100.00	52.32 ± 0.14	17.40–100.00
ALH (µm)	3.36 ± 0.02	1.50–6.40	1.10 ± 0.00	0.00–2.80	2.61 ± 0.01	0.80–4.00	1.83 ± 0.01	0.40–3.50
BCF (Hz)	36.67 ± 0.31	0.00–73.50	14.39 ± 0.10	0.00–52.40	35.29 ± 0.19	0.00–72.10	28.39 ± 0.14	0.00–70.80
**n (%)**	1776 (9.97%)		5609 (31.50%)		4339 (24.36%)		6085 (34.17)	

## Data Availability

All data is contained within the article.
